# An injectable magnesium-coordinated phosphate chitosan-based hydrogel loaded with vancomycin for antibacterial and osteogenesis in the treatment of osteomyelitis

**DOI:** 10.1093/rb/rbae049

**Published:** 2024-05-25

**Authors:** Peng Zhang, Tiehua Wang, Junyu Qian, Haotian Qin, Peng Liu, Ao Xiong, Anjaneyulu Udduttula, Deli Wang, Hui Zeng, Yingqi Chen

**Affiliations:** Department of Bone & Joint Surgery, National & Local Joint Engineering Research Center of Orthopaedic Biomaterials, Peking University Shenzhen Hospital, Shenzhen 518036, China; Internal Medicine, Shenzhen New Frontier United Family Hospital, Shenzhen 518031, China; Department of Bone & Joint Surgery, National & Local Joint Engineering Research Center of Orthopaedic Biomaterials, Peking University Shenzhen Hospital, Shenzhen 518036, China; Department of Bone & Joint Surgery, National & Local Joint Engineering Research Center of Orthopaedic Biomaterials, Peking University Shenzhen Hospital, Shenzhen 518036, China; Department of Bone & Joint Surgery, National & Local Joint Engineering Research Center of Orthopaedic Biomaterials, Peking University Shenzhen Hospital, Shenzhen 518036, China; Department of Bone & Joint Surgery, National & Local Joint Engineering Research Center of Orthopaedic Biomaterials, Peking University Shenzhen Hospital, Shenzhen 518036, China; Centre for Biomaterials, Cellular and Molecular Theranostics (CBCMT), Vellore Institute of Technology (VIT), Vellore 632014, India; Department of Bone & Joint Surgery, National & Local Joint Engineering Research Center of Orthopaedic Biomaterials, Peking University Shenzhen Hospital, Shenzhen 518036, China; Department of Bone & Joint Surgery, National & Local Joint Engineering Research Center of Orthopaedic Biomaterials, Peking University Shenzhen Hospital, Shenzhen 518036, China; Department of Bone & Joint Surgery, National & Local Joint Engineering Research Center of Orthopaedic Biomaterials, Peking University Shenzhen Hospital, Shenzhen 518036, China

**Keywords:** injectable chitosan hydrogel, vancomycin loading and release, antibacterials property, osteogenic promotive property, osteomyelitis treatment

## Abstract

Microbial infections of bones, particularly after joint replacement surgery, are a common occurrence in clinical settings and often lead to osteomyelitis (OM). Unfortunately, current treatment approaches for OM are not satisfactory. To address this issue, this study focuses on the development and evaluation of an injectable magnesium oxide (MgO) nanoparticle (NP)-coordinated phosphocreatine-grafted chitosan hydrogel (CMPMg-VCM) loaded with varying amounts of vancomycin (VCM) for the treatment of OM. The results demonstrate that the loading of VCM does not affect the formation of the injectable hydrogel, and the MgO-incorporated hydrogel exhibits anti-swelling properties. The release of VCM from the hydrogel effectively kills *S.aureus* bacteria, with CMPMg-VCM (50) showing the highest antibacterial activity even after prolonged immersion in PBS solution for 12 days. Importantly, all the hydrogels are non-toxic to MC3T3-E1 cells and promote osteogenic differentiation through the early secretion of alkaline phosphatase and calcium nodule formation. Furthermore, *in vivo* experiments using a rat OM model reveal that the CMPMg-VCM hydrogel effectively kills and inhibits bacterial growth, while also protecting the infected bone from osteolysis. These beneficial properties are attributed to the burst release of VCM, which disrupts bacterial biofilm, as well as the release of Mg ions and hydroxyl by the degradation of MgO NPs, which inhibits bacterial growth and prevents osteolysis. Overall, the CMPMg-VCM hydrogel exhibits promising potential for the treatment of microbial bone infections.

## Graphical abstract

**Figure:**
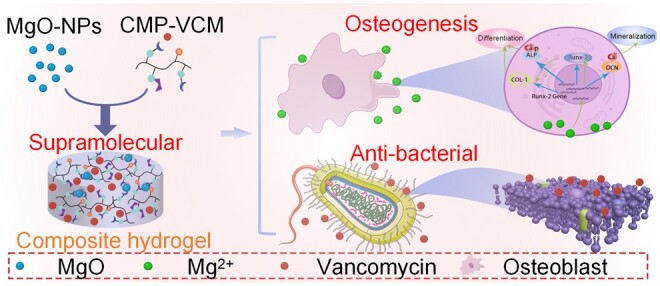

## Introduction

Osteomyelitis (OM), usually caused by microbial infection in bones, is common in clinics, such as after surgical instrument implantation. The pathogenic bacteria for OM are *Staphylococcus aureus* (*S.aureus*). In opening orthopaedic trauma, more than 20% of patients will suffer from OM, which leads to osteolysis, bone destruction and sequestrum formation [1]. The clinic treatment method for OM is multiple surgical debridement and long-term and high-dose use of antibiotics in local sites or systemic administration [2], but they suffer from inefficiency, high cost, high recurrence and morbidity and/or even serious bacterial resistance. One of the standard methods in clinics is to use antibiotic-loaded poly(methylmethacrylate) (PMMA) bone cement for the local release of drugs against OM, but suffer from limited antibiotic drug loading and releasing, an unamiable polymerization environment for drugs, and a second surgery to remove the non-biodegradable PMMA [3]. In the last decades, with the development of biomaterials and regenerative medicine, newly designed biomaterials with outstanding anti-bacterial, biodegradability and osteogenic promotive properties should be an alternative way to circumvent these issues.

In the OM treatment, the most important attribute of biomaterials should be their excellent bactericidal property in the initial stage to break the bacterial film formed by OM, and in the long-term implantation process, the biomaterials should also possess the antibacterial property to inhibit the growth and reproduction of bacteria. Meanwhile, the other properties are also important to the OM treatment. To this end, various kinds of biomaterials loaded (or not) with different antimicrobial agents or antibiotics have been designed and developed to address the challenges during the OM treatment. The reported biomaterials included biodegradable metals [4], natural antimicrobial peptides-based biomaterials [5], biodegradable polymers [6], chitosan (CS) [7], hydrogels [8], nanoparticles (NPs) [9], nanohydroxyapatite/collagen granules [10], gelatin microspheres [11] and others. Among these options, hydrogels, especially those made from natural polymers, have shown great promise in OM treatment as essential attributes in biocompatibility, biodegradability, antibacterial drug loading and controlled release. CS, a kind of natural polymer, is extracted from the shells of crustaceans and possesses the desirable properties in biodegradability, biocompatibility and antimicrobial properties. CS and CS derivates have been largely used as matrix materials for bone regeneration [12] and drug loading and release carrier materials [13]. However, the insoluble nature of CS limits its further biomedical applications. Numerous CS derivates have been developed, such as carboxymethyl CS [7], methacryloyl CS [14] and phosphate CS [12], for tissue regeneration as the water-soluble attribute of these CS derivates. The antibacterial mechanism of CS is that the abundant positive charged amino groups in the CS backbone can electrostatically combine with negatively charged bacteria, and then the CS accumulates on the wall, followed by destruction of the bacterial membrane, which then results in the death of bacteria. Grafting with other molecules in CS would consume the amino groups, which compromises the antibacterial efficacy of CS. The intrinsic antibacterial properties of CS, especially its derivates, are far from satisfactory in OM treatment. Therefore, loading and controlled release of antibiotic agents for CS-based hydrogels is of importance for OM treatment. To our best knowledge, almost no study has been reported to endow the dynamically varying antibacterial properties and the osteogenic promotive properties.

Magnesium oxide (MgO) NPs are a kind of important magnesium-contained compound that can be degraded in a physiological environment or chloride-contained aqueous solution to produce magnesium ions and hydroxyl ions. Previous studies confirmed that Mg ions combined with hydroxyl ions possessed antibacterial properties [15], and Mg ions in suitable concentrations have osteogenic promotive properties [16], especially in the alkalescent environment [17]. Recent studies have been reported to incorporate MgO NPs into CS-based hydrogel systems [12], polymeric scaffolds [18, 19] and other composite scaffolds [20] to improve the mechanical properties and angiogenic and osteogenic promotive properties. Our previous study verified that the MgO NPs can combine with phosphate groups in phosphocreatine-grafted CS to form injectable hydrogel via metal-organic coordination [12], and in this study, the osteogenic and angiogenic properties of this hydrogel have been investigated and confirmed. However, the antibacterial properties of this hydrogel may need to be greatly improved, especially in the OM treatment. Vancomycin (VCM), as a kind of glycopeptide antibiotic, has antibacterial ability against gram-positive bacteria, such as SA, via interfering with the production of bacterial cell walls [21]. VCM has been utilized for OM treatment in clinics via local and systemic administration methods. However, local delivery of high-concentration VCM will restrain bone growth and easily cause bone loss at the local site, which requires multiple local drug deliveries. Therefore, the design and development of a biomaterial system to load and control the release of VCM will be desirable for OM treatment.

Herein, the water-soluble VCM was directly mixed into a phosphocreatine-grafted CS solution, and then MgO NPs were added to this mix solution to form the VCM-loaded magnesium-phosphate coordinated hydrogel (CMPMg-VCM). The burst release of VCM in the initial stage will break the bacterial film, and in the long-term implantation process, the controlled release of magnesium ions and hydroxyl groups will promote new bone growth and inhibit the propagation of bacteria. The physicochemical properties, including rheological property, anti-swelling property, compressive property, antibacterials property to SA, cytotoxicity to MC3T3-E1 cells and *in vitro* osteogenic promotive property to MC3T3-E1 cells, were systematically investigated. Finally, the rat tibia OM model was created by injecting a large amount of *S.aureus* into the medullary cavity, and then the hydrogels were implanted into the medullary cavity to investigate the OM treatment efficacy and *in vivo* osteogenic promotive properties.

## Materials and methods

### The synthesis of phosphate-functionalized methacryloyl chitosan (CMP)

We synthesized the phosphocreatine (PS, C104990, Aladdin, Shanghai, China) functionalized methacryloyl CM polymer according to the protocol in the previous study [12]. Specifically, 1 g CS (CS, 448869, Sigma, USA) was dissolved in 100 ml of 1% acetate (A116166, Aladdin, Shanghai, China) water solution, and then 1.1 ml of methacrylic anhydride (MA, 276685, Sigma, USA) was gently added to this solution. After stirring at room temperature for at least 12 h, the 100 ml reacted CS solution was slowly added into 50 ml of 2.7 g PS mixed with 0.9 g of 1-ethyl-3-(3-dimethylaminopropyl)-carbodiimide (EDC, 03450, Sigma, USA) and 0.3 g N-hydroxysuccinimide (NHS, 56480, Sigma, USA) solution. After 24 h of stirring at room temperature, the reacted solution was dialyzed in DI water for 7 days and then lyophilized to obtain the fl^oc^culent-like CMP.

### Hydrogel fabrication

We purchased Vancomycin HCl (VCM) from MeilunBio (containing approximately 900 g/mg, MB1260, Dalian, China). MgO NPs (the average diameter was ca. 20 nm according to the vendor) were purchased from XFNANO (100369, Nanjing, China). The synthesized CMP polymer was dissolved in DI water at a concentration of 20 mg/ml. Then, different amounts of VCM were added to a 5-ml CSMP solution to make different concentrations (0.1, 0.5, 1 and 5 mg/mL) of VCM-contained solutions, followed by adding 50 μl of 10 mg MgO nanoparticle water solution to this solution. As VCM was water-soluble and could be entirely added to the pre-gel solution, the loading capacity was the dissolution limit of VCM in this pre-gel solution in theory. After being mixed well, the VCM-incorporated injectable hydrogels were formed. The different concentrations of VCM-incorporated hydrogels were named CMPMg-VCM (1, 5, 10 and 50), and the sole MgO-incorporated injectable hydrogel without VCM was named CMPMg. The chemical hydrogel without MgO nanoparticles and VCM was synthesized with the method described in the reported study [14].

### Characterization

The hydrogels were lyophilized, and after being gold sprayed, the surface morphology was observed with scanning electron microscopy (SEM, JEOL, JSM-7401F, Tokyo, Japan), and the elemental distribution was detected with the energy-dispersive spectrometer (EDS) equipped in the SEM. We measured the rheological properties of hydrogels using a rheometer (ESCALAB, 250Xi). The disc sample (20 mm in diameter and 1 mm in height) was placed on the testing plate, and then a time sweep was performed with a strain of 10%, and the frequency ranged from 0.1 to 10 Hz.

The swelling ratio of hydrogels was performed by immersing the hydrogels in DI water solution. The cylindrical hydrogel samples (8 mm in diameter and 10 mm in height) were immersed in DI water, and after swelling in DI water for 24 h the samples were taken out and imaged with a digital camera. Meanwhile, we measured the diameter and height of the swollen hydrogels using a vernier caliper and calculated the volume as *V*_1_. The volume change ratio was calculated with the following formula: *V*_1_/*V*_0_ × 100%, the *V*_0_ was calculated before the immersion. Four parallel samples were performed for statistical analysis.

We tested the compression property of hydrogels using a material testing machine (Instron-E3000, Norwood, MA). We performed the compression testing on a cylindrical hydrogel sample (8 mm in diameter and 10 mm in height). The compression speed was 5 mm/min. Each group received four parallel samples. We obtained the compressive strength and modulus from the compressive strain vs. stress curves.

A cylindrical hydrogel with 8 mm in diameter and 10 mm in height was immersed in a 3 ml PBS solution in an air bath with the rocking state at 37°C. At the set time point, all the immersed solution was collected to detect the VCM concentration with an atomic absorption spectrophotometer (AA-7003, EWAI, Beijing, China), and a new 3-ml PBS solution was added to each sample. We obtained the accumulated VCM release curves for each sample. After immersion, the accumulated release ratio of VCM was calculated according to the following formula: *M*_1_/*M*_0_ × 100%, the *M*_1_ was the quality of the accumulated released VCM, and the *M*_0_ was the quality of the loaded VCM. The quality of the loaded VCM was calculated from the added VCM concentration and the volume of the immersed hydrogel. For the magnesium ion release assay, a cylindrical hydrogel sample (8 mm in diameter and 6 mm in height) was immersed in a 3-ml PBS solution in an air bath with the rocking state at 37^°^C. At the set time point, the immersed PBS solution was collected to detect the magnesium ion concentration with the same atomic absorption spectrophotometer, and the newly prepared 3 ml PBS solution was added. Finally, the magnesium ion release curves were obtained. We conducted this investigation on four parallel samples.

### Antibacterial properties of hydrogels

*Staphylococcus aureus* (CMCC(B)26003, HuanKai Microbial, Guangzhou, China) was cultured in soy casein culture medium (HuanKai Microbial) at 37^°^C in the rocking state. After the optical density (O.D.) of the medium reached 0.5, the bacterial medium solution was used for further antibacterial property testing investigations.

The bacterial medium solution was carefully and uniformly coated on an agar culture dish, and then the sterilized lyophilized hydrogel samples were placed on the culture dish. The dish was cultured in an incubator at 37°C with 5% CO_2_ for 24 h. After that, the images for each dish were taken, and the diameter of the inhibitory zone in each sample was measured with a vernier caliper. Three parallel samples for each group were performed for statistical analysis, and the representative images were used for display.

One sterilized lyophilized hydrogel sample (8 mm in diameter and 10 mm in height) was immersed in 3 ml of PBS and then placed in an air bath shaker at 37^°^C with a shaking rate of 100 rpm. At each set time point (days 3, 6, 9 and 12), half of the immersed PBS (1.5 ml) solution was taken out of each sample for antibacterial property testing, and 1.5 ml of fresh PBS was added to each sample for further immersion. About 1.5 ml of the samples’ immersed PBS solution was mixed with 1.5 ml of the bacterial medium solution (the same as the aforementioned), and then this mixed solution was cultured in an air bath shaker at 37°C with a shaking rate of 100 rpm for 24 h. After that, 1 ml of this mixed solution was taken for O.D. detection at 600 nm; 1 ml of the mixed solution was carefully and uniformly coated on an agar culture dish and after 24 h of culture in an incubator (37°C, 5% CO_2_), the images for each dish were taken.

### *In vitro* cell culture study

We used the murine calvaria pre-osteoblast cells (MC3T3-E1, ATCC CRL-2594, USA) to assess the cell viability and osteogenic promotive properties for the samples. MC3T3-E1 cells were cultured in minimum essential medium alpha (α-MEM, Hyclone, USA), including 10% FBS and 1% penicillin/streptomycin. We lyophilized the cylindrical hydrogel samples (8 mm in diameter and 1.5 mm in height) and sterilized them under ultraviolet (UV) irradiation for 1 h before using them for cell study.

About 1 ml of cell suspension solution with 5 × 10^3^ cells was seeded into a well in a 24-well plate, and after 24 h of culture, the sterilized lyophilized hydrogel sample was placed in the chamber in a transwell, which was placed onto each well. Every three days, we refreshed the medium. For the live/dead cells assay, after 3 and 5 days of culture, the medium was removed from each well, and the live/dead cells were stained with the live/dead staining kit (Calcein-AM/PI double stain kit, Shanghai, China) according to the instructions. After staining, the cells were observed using fluorescence microscopy (OLYMPUS-IX51, Japan). For cell viability assay, after 3- and 5-days culture, the medium was removed from each well, and 300 μl of medium containing 10% cell counting kit-8 solution (CCK-8, Sigma-Aldrich, USA) was added to each well. After incubating in a cell culture incubator for 1 h, 200 μl of solution from each well was carefully transferred to a new 96-well plate, and then the O.D. was detected at 450 nm using a microplate reader. The cell viability was calculated according to the following: [(*A*_s _− *A*_b_)/(*A*_c _− *A*_b_)] × 100%, where *A*_s_ was the O.D. of tested samples, *A*_b_ and *A*_c_ were the O.D. of the positive and negative control.

For the alkaline phosphatase (ALP) activity and alizarin red assay, 1 ml of cell suspension with 5 × 10^4^ cells was seeded into a well in a 24-well plate. After 24 h of culture, the sterilized lyophilized hydrogel sample was placed in the chamber in a transwell, which was placed onto each well, and the medium was changed to osteogenic promoting medium, which contained β-glycerophosphate (1 mol l^−1^; Sigma, USA), ascorbate acid (50 mmol l^−1^; Sigma, USA), and dexamethasone (1 mmol l^−1^; Sigma, USA). Every three days, we refreshed the medium. After 7- and 14-day cultures, the medium was removed, and the cells were fixed with 4% paraformaldehyde. After being washed with PBS three times, the cells were stained with the BCIP/NBT alkaline phosphatase kit (Beyotime, China) according to the instructions of the kit. For the ALP activity assessment, after 7- and 14-day culture, the medium was removed, and the cells were lysed with 1% Triton. The cell lysate was used for the ALP activity assay using an alkaline phosphatase kit (Nanjing Jiancheng, China) according to the instructions of the kit. We used a bicinchoninic acid protein assay kit (BCA, Jiancheng Bioengineering Institute, Nanjing) to detect the protein concentration. We obtained the ALP activity using the alkaline phosphatase kit. For the alizarin red assay, after 21 days of culture, the medium was removed and the cells were rinsed with PBS three times. Then, 200 μl of alizarin red staining (ARS) solution (Solarbio Science & Technology Co., Ltd, Beijing) was added to each well, and after 20 min of incubation at room temperature, the ARS solution was removed. The cells were rinsed with deionized water several times until no stained solution was observed. The cells were imaged with optical microscopy (MM6, Leitz Company). For the quantitative analysis, the stained dye was dissolved with 10% hexadecylpridinium chloride (Sigma, USA), and the O.D. of the solution was detected at 562 nm. For the cell culture study, four parallel samples were performed for statistical analysis.

### *In vivo* study

We purchased the female Sprague Dawley rats, weighing approximately 220 g, from the Guangdong Medical Laboratory Animal Center in Guangzhou, China. All the surgical procedures were followed the instructions approved by the Institutional Animal Care and Use Committee of Peking University Shenzhen Hospital (ethical approval No. 2022-164). The rats were separated into five groups, and each group had six rats. After anaesthesia with isoflurane, the hair around the knee was shaved. Then, the skin and the muscle around the lateral patella were cut and separated, and the tubercle of the tibia was exposed. Then, a hole (1 mm in diameter) was made in the tubercle of the tibia using a dental drill. About 10 μl 1 × 10^8^ CFU/ml bacterial suspension (in PBS solution) was added into the tibial medullary cavity, followed by the implantation of different samples. The implanted hydrogel samples were CMP, CMPMg and CMPMg-VCM (50), and the CMPMg-VCM (50) was shorted as CMPMg-VCM in the main figures display. The negative control was injected with a bacterial suspension, and the positive control was implanted with a sterilized PBS solution. Subsequently, we sealed the hole with bone wax, layer by layer, covering the wound. After 3- and 6-week implantation, the rats were euthanasiad, and the tibia was obtained for further investigation. The organs were obtained for *in vivo* biosafety investigations. The tibia (after 6-week implantation) was immersed in a 10-ml PBS solution, which was placed in an ultrasonic cleaning machine (50 Hz, B3500SMT, China) for 10 min. Then, after the immersed PBS solution was diluted 20 times, 20 μl of this PBS bacterial suspension was carefully and uniformly coated on an agar culture dish, which was placed in a culture medium for 24 h in an incubator (37°C, 5% CO_2_). Then, the images for the plate cultured with bacterial suspension were taken, and the colony-forming units (CFU) were calculated.

We fixed the obtained tibia in paraformaldehyde. The three-dimensional computed tomography of the tibia was scanned with a Vivo 80 micro-CT system (Scanco, Switzerland). The region of interest (ROI) for bone-related parameter analysis was 100 scanning surfaces, which are from the bone scale line to the distal tibia. The bone-related parameters include bone mineral density, the ratio of bone volume to tissue volume and trabecular numbers.

The fixed tibias were immersed in a 10% EDTA solution for two months to undergo decalcification. Then, the tibias were embedded in paraffin, and cross sections of tibia were cut at 5 μm in thickness for haematoxylin and eosin (H&E), Masson staining and Gram’s staining according to the instructions. We evaluated the semi-quantitative histological scoring analysis for the inflammation in H&E staining from the reported studies [22, 23], summarizing and presenting the scores for capsule thickness, qualitative bone reaction, inflammatory response and ingrowth. The obtained organs were fixed with paraformaldehyde and then embedded in paraffin for tissue section, followed by H&E staining.

## Results

### Fabrication, rheological and compressive properties of CMPMg-VCM hydrogel

**Figure 1.**
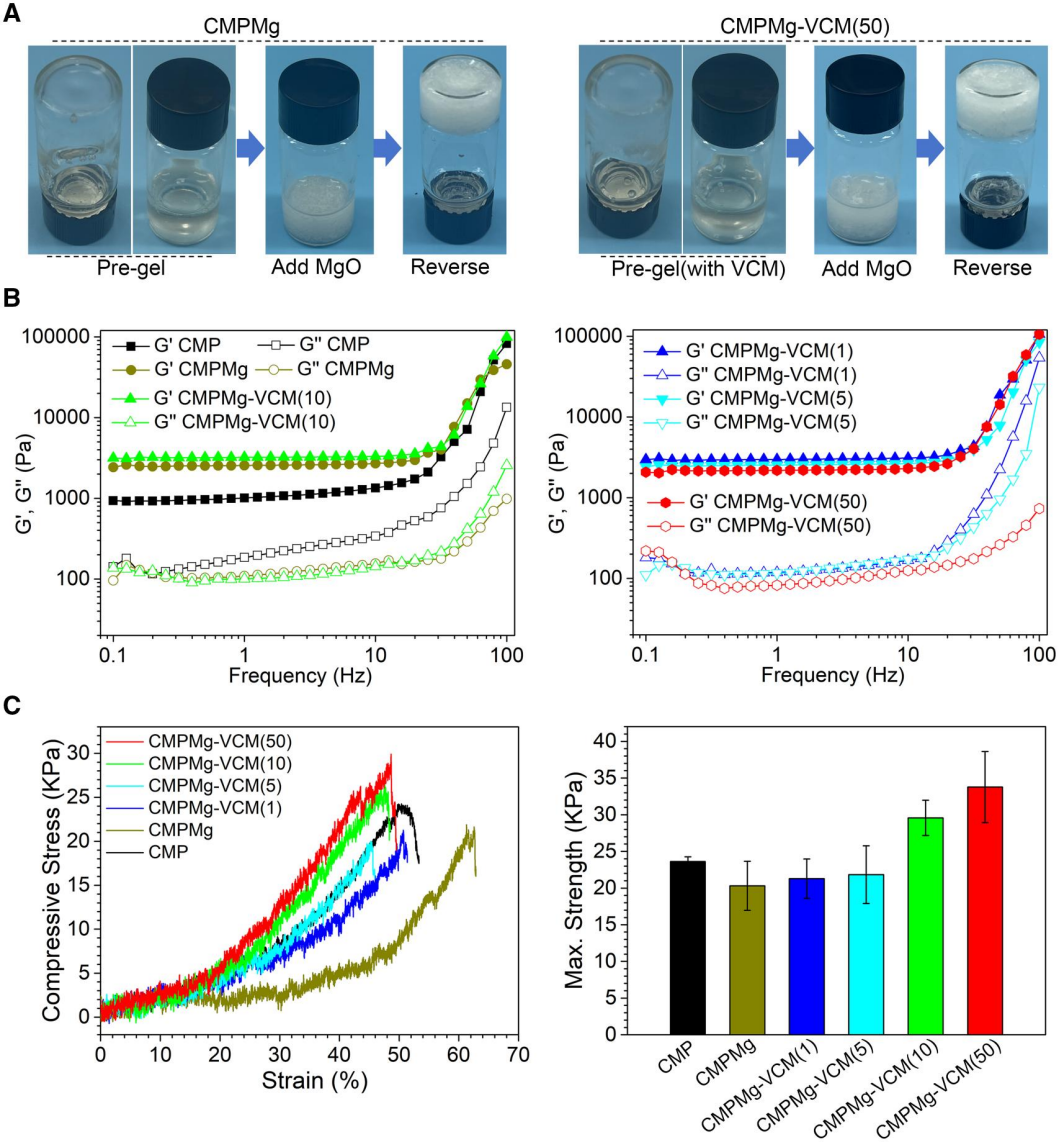
Preparation and rheological properties of injectable hydrogels. (**A**) Gelation process of CMPMg-VCM hydrogel versus CMPMg hydrogel. (**B**) Frequency sweeping of viscous moduli *G*″ and elastic moduli *G*′ of CMPMg-VCM hydrogel versus CMPMg hydrogel from 0.1 to 100 Hz under 1% stain at 37°C. (**C**) Representative compressive stress vs. strain curves, and maximum strength of hydrogels obtained from stress vs. strain curves (*n* = 4).

Figure 1A shows the tube inversion test to investigate the fabrication of CMPMg-VCM hydrogel. The phosphocreatine-grafted CS with or without VCM would form a hydrogel after the addition of MgO nanoparticles. From the rheological property result in Figure 1B, all the injectable hydrogels, including CMPMg, CMPMg-VCM (1, 5, 10 and 50), have a higher elastic moduli *G*′ than that of chemically crosslinked CMP hydrogel. All the injectable hydrogels had similar elastic moduli *G*′, indicating the inclusion of VCM in the hydrogel would not weaken the hydrogel. The compressive property results of hydrogels are presented in Figure 1C. The CMPMg-VCM (10 and 50) hydrogel had a higher compressive strength than those of CMP and CMPMg hydrogels.

### Anti-swelling property of hydrogels

**Figure 2.**
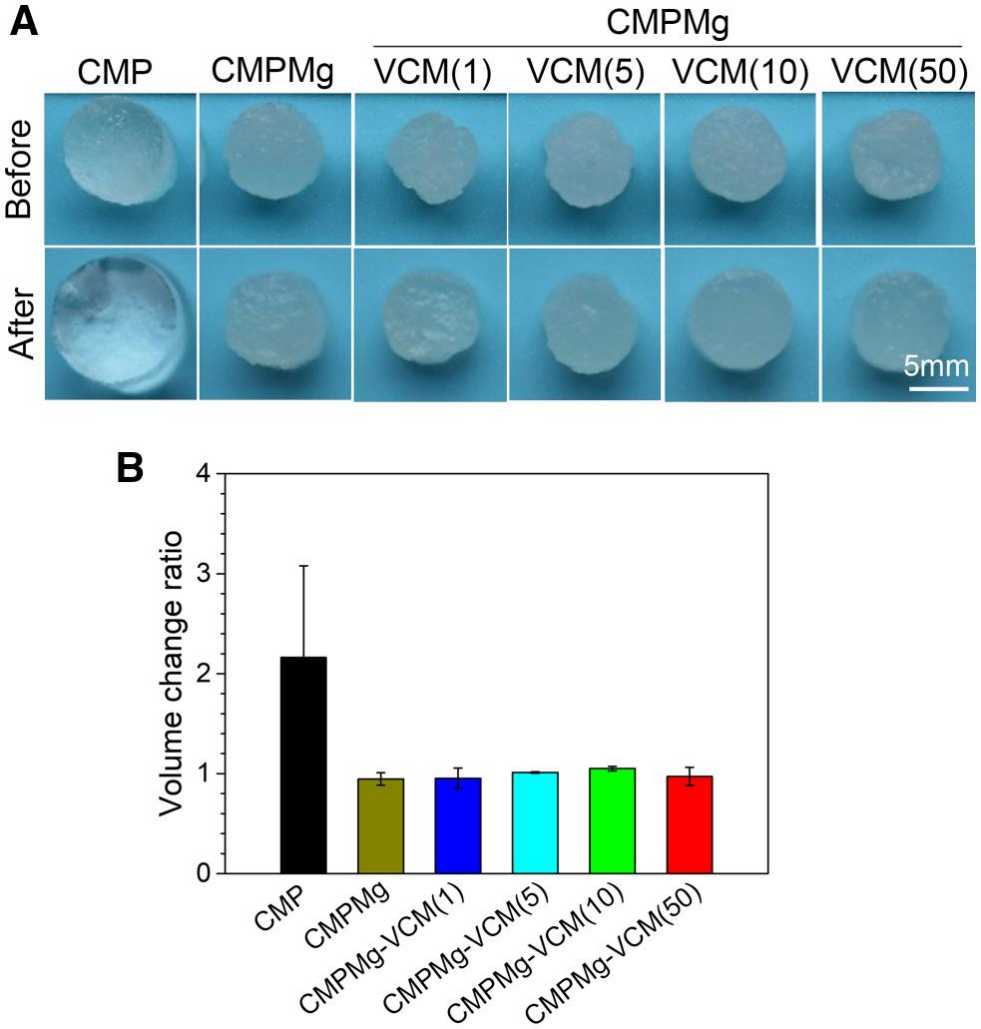
Anti-swelling properties of hydrogels when immersed in DI water. (**A**) The macroscopical images for the hydrogels, and (**B**) the volume change ratio of hydrogels before and after swollen in DI water for 24 h (*n* = 4, *P < 0.05).

Figure 2 shows the anti-swelling properties of hydrogels immersed in DI water. From the macroscopical images in Figure 2A, all the injectable hydrogels showed no obvious change between before and after swelling in DI water. The statistical analysis result for the volume change ratio in Figure 2B showed the same tendency with macroscopical observation.

### Surface morphology and EDS analysis

**Figure 3.**
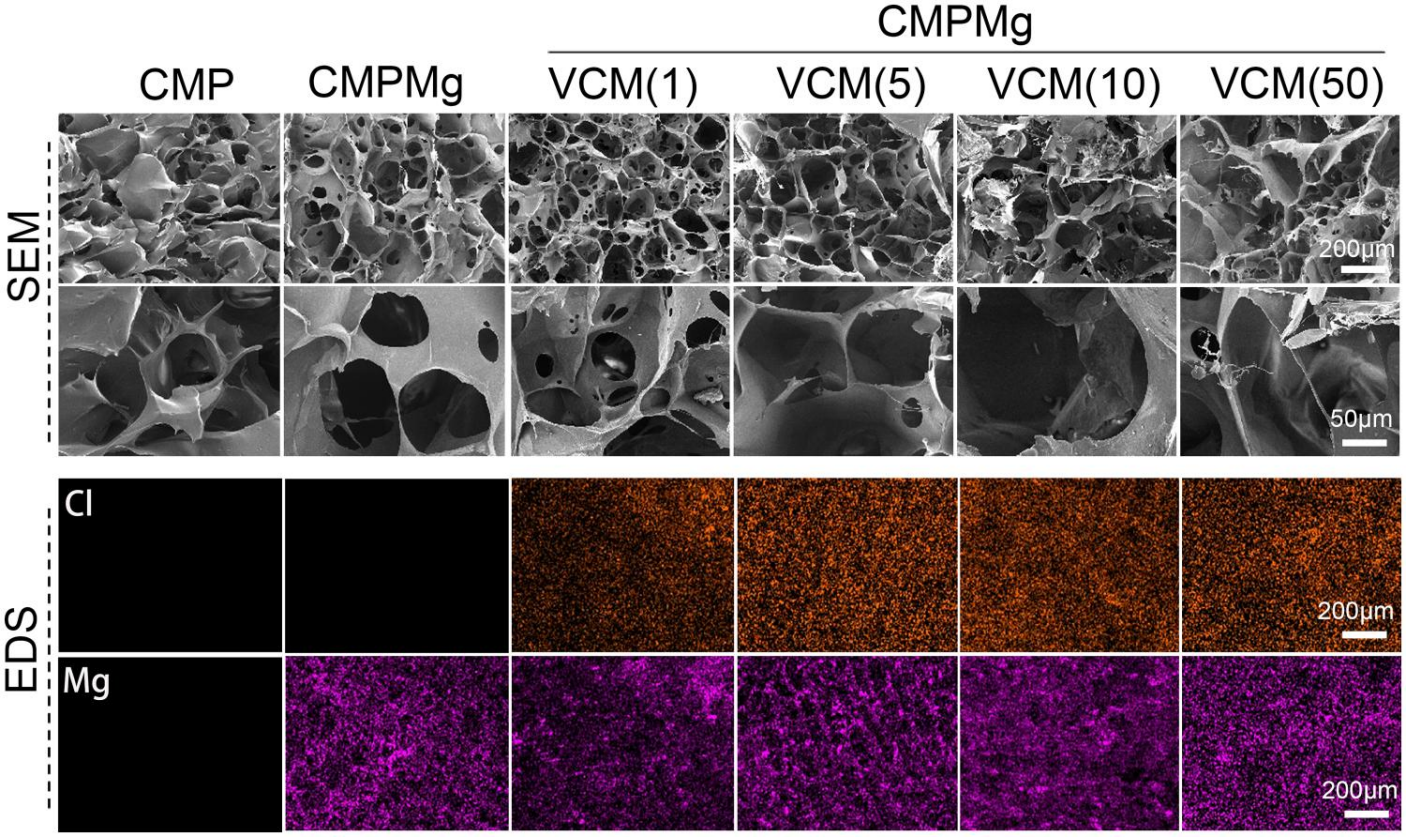
Surface SEM images and surface EDS mapping for the lyophilized hydrogels.

Figure 3 presents the surface morphology and EDS results for the lyophilized hydrogels. All the CMPMg samples showed porous-like morphology; the pore size was around 100–150 μm, and the same small holes were displayed in the thin pore walls. The chemical bond CMP hydrogel also presented a porous-like morphology, but no small holes were observed in the pore walls. In the EDS results, the element Cl was from the VCM, and the element Mg was from MgO nanoparticles. As shown, all the VCM-loaded hydrogels showed the element Cl and all the MgO-incorporated hydrogels displayed the element Mg, indicating the existence of these components (VCM and MgO) in the CMPMg-VCM hydrogel.

### VCM and magnesium ions release profile

**Figure 4.**
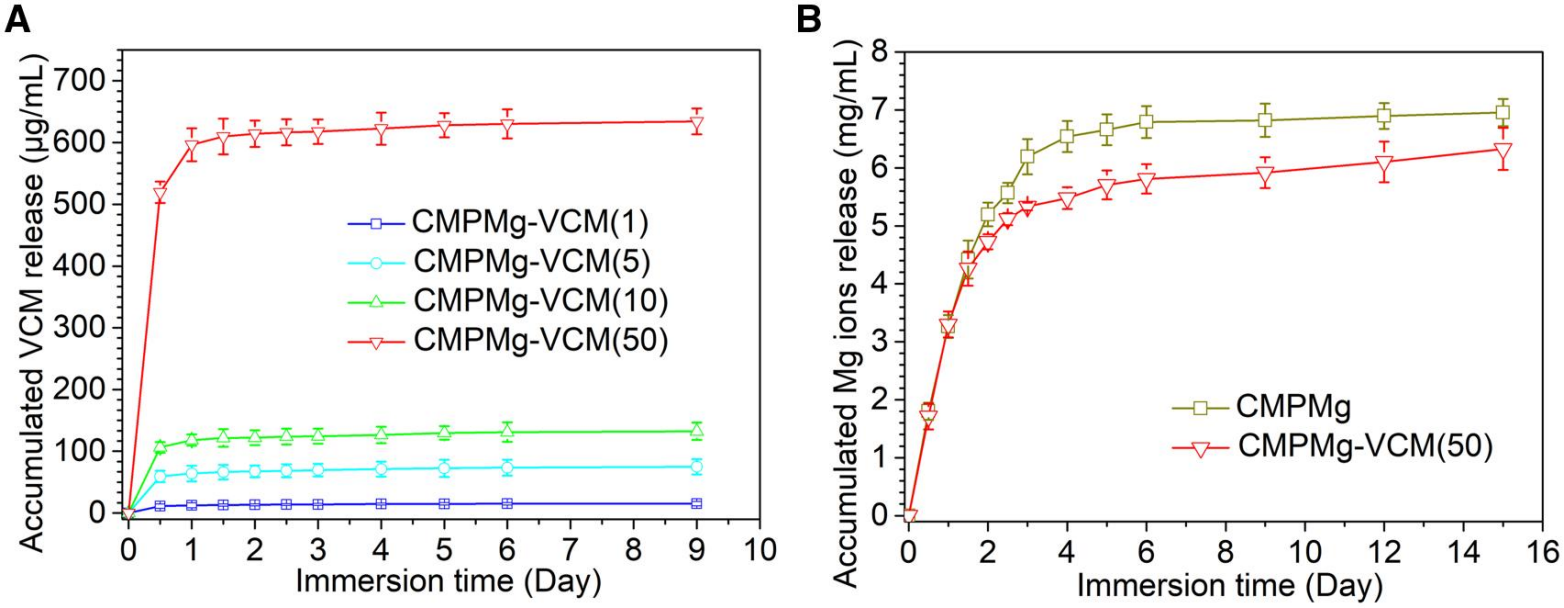
The accumulated VCM release for the hydrogels immersed in PBS solutions at 37 ± 0.5°C in the rocking state.

Figure 4 shows the VCM and magnesium ions release profile of hydrogels when immersed in PBS. The VCM was released quickly from the hydrogels in the initial immersion stage (up to 3 days), and then the remaining VCM in the hydrogel was slowly released for several days (9 days in this study). The higher concentration of VCM in hydrogels released more VCM. We calculated the accumulated release ratio of VCM for CMPMg-VCM (1, 5, 10 and 50) as 75.7%, 79.1%, 89.5% and 91.0%, respectively, based on the released curves. More than 75% of the loaded VCM was released from the hydrogels after immersion. The magnesium ions release results for CMPMg and CMPMg-VCM (50) showed that magnesium ions were released fast in the initial 4 days of immersion and then slowly until 15 days. In the middle immersion stage (from 3 to 12 days), the magnesium ions release for CMPMg was larger than that of CMPMg-VCM (50), indicating the included VCM prohibited the magnesium ions release in this period, but after 15 days of immersion, the released Mg ions amount for these hydrogels was similar.

### Antibacterial properties of hydrogels

**Figure 5.**
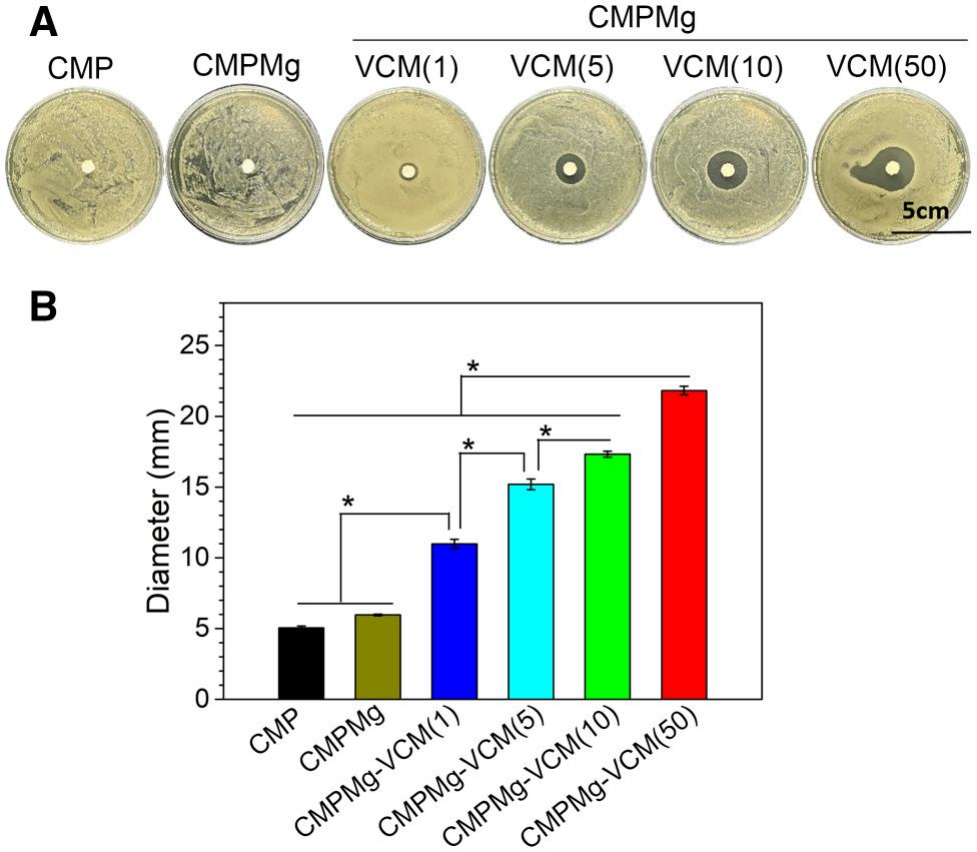
Antibacterial property of hydrogels to *S.aureus*. (**A**) Macroscopical images for the bacteriostatic ring test, and (**B**) statistically analysis result for the bacteriostatic ring diameter (*n* = 4, *P < 0.05).

**Figure 6.**
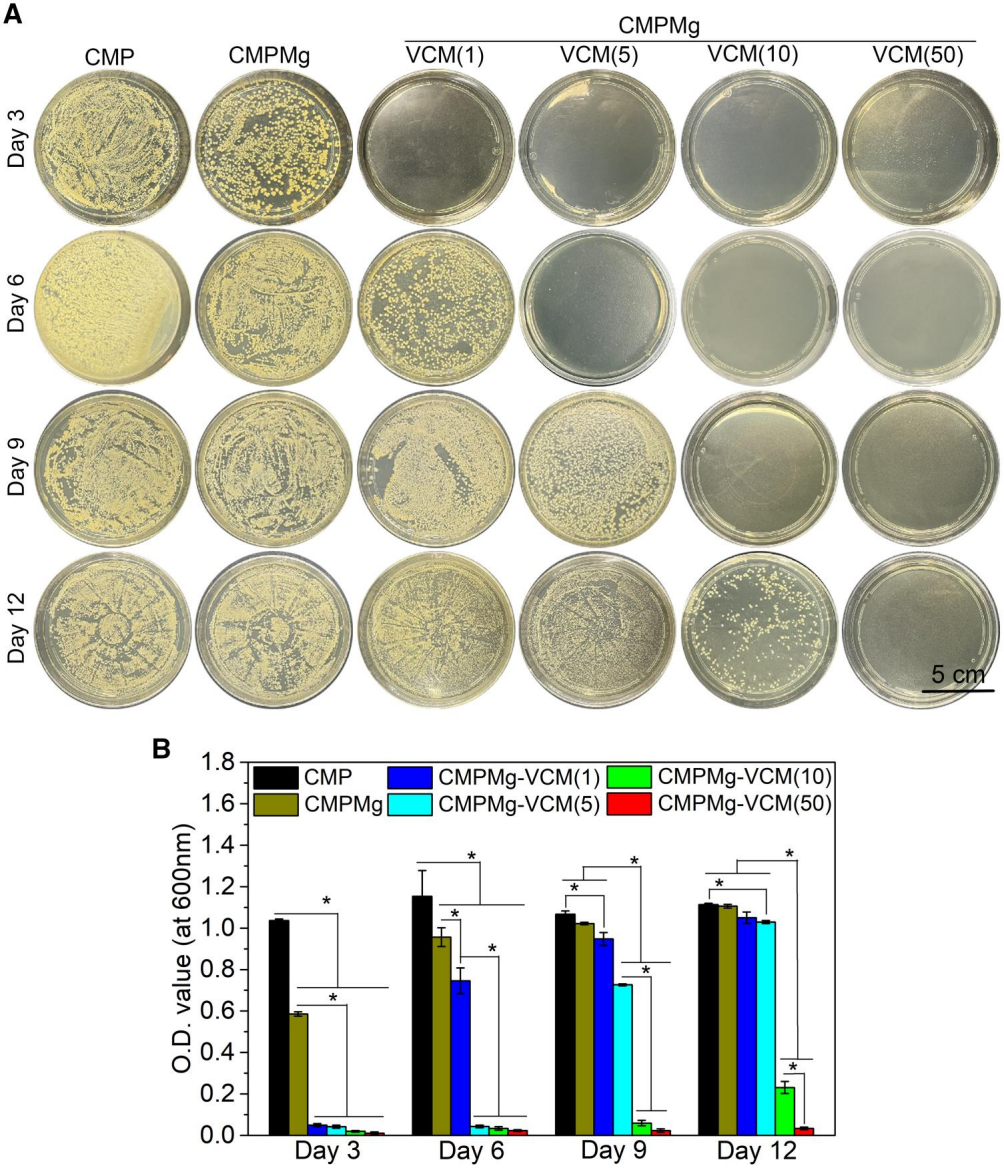
Turbidity assay for the *S.aureus* cultured with PBS solution immersed with hydrogels for 3, 6, 9 and 12 days. (**A**) Macroscopical images for the *S.aureus* colony after the immersed PBS and SA bacterial medium mixed solution cultured on agar culture dish for 24 h, and (**B**) the O.D. value for the mixed solution cultured in an air bath shaker at 37°C with the shaking rat of 100 rpm for 24 h (*n* = 4, *P < 0.05).

*Staphylococcus aureus* is the most prevalent infectious bacteria in OM. Thus, the antibacterial properties of hydrogels for SA were investigated, and the results are presented in Figures 5 and 6. From Figure 5A, the bacteriostatic ring diameter for VCM-loaded hydrogels was larger than that of CMPMg and CMP hydrogels, and among these VCM-loaded hydrogels, the larger VCM-loaded hydrogel had a bigger bacteriostatic ring. The statistical analysis result in Figure 5B showed the same tendency for macroscopical observation as in Figure 5A. Further, to simulate the *in vivo* implantation environment, the antibacterial properties of PBS solution after being immersed with hydrogels for different times were investigated. In Figure 6A, after 3 days of immersion, no obvious bacterial colony was displayed on the VCM-loaded hydrogels (CMPMg-VCM (1, 5, 10 and 50)) immersed solutions, and the CMP and CMPMg immersed solutions presented plenty of bacterial colonies. With the immersion time up to 12 days, the bacterial colony was gradually presented in low concentrations of VCM-loaded hydrogels (CMPMg-VCM (1, 5 and 10)) with increased immersion time, and the highest concentration of VCM-loaded hydrogel (CMPMg-VCM (50)) still showed no obvious bacterial colony after 12 days of immersion. The statistical analysis results for the turbidity test in Figure 6B confirmed this tendency in Figure 6A.

### *In vitro* cell culture study

**Figure 7.**
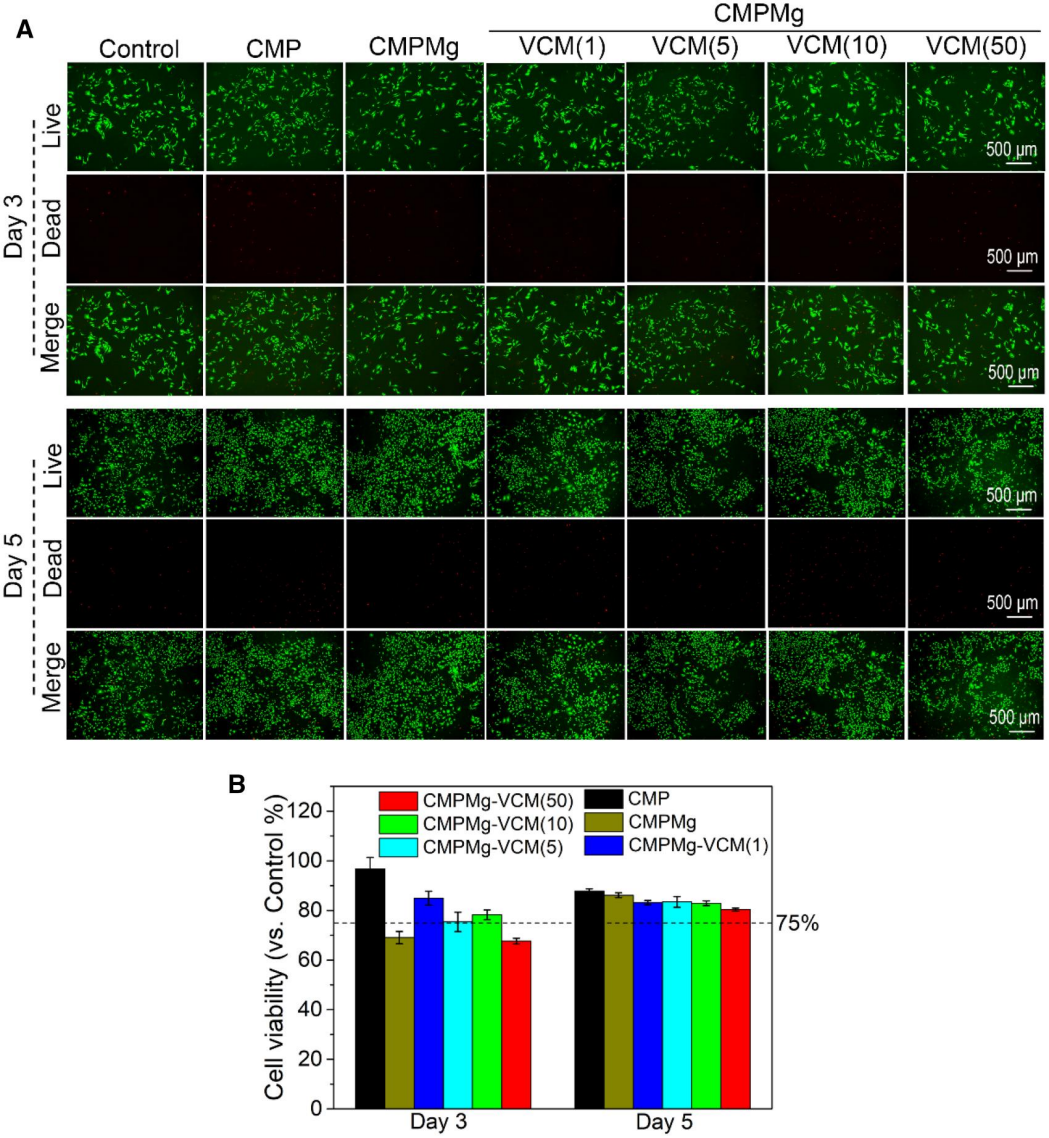
Cytotoxicity of hydrogels when cultured with MC3T3-E1 cells. (**A**) Representative fluorescent images for live/dead cell when cultured with hydrogels for 3 days, and (**B**) CCK8 results (*n* = 4).

**Figure 8.**
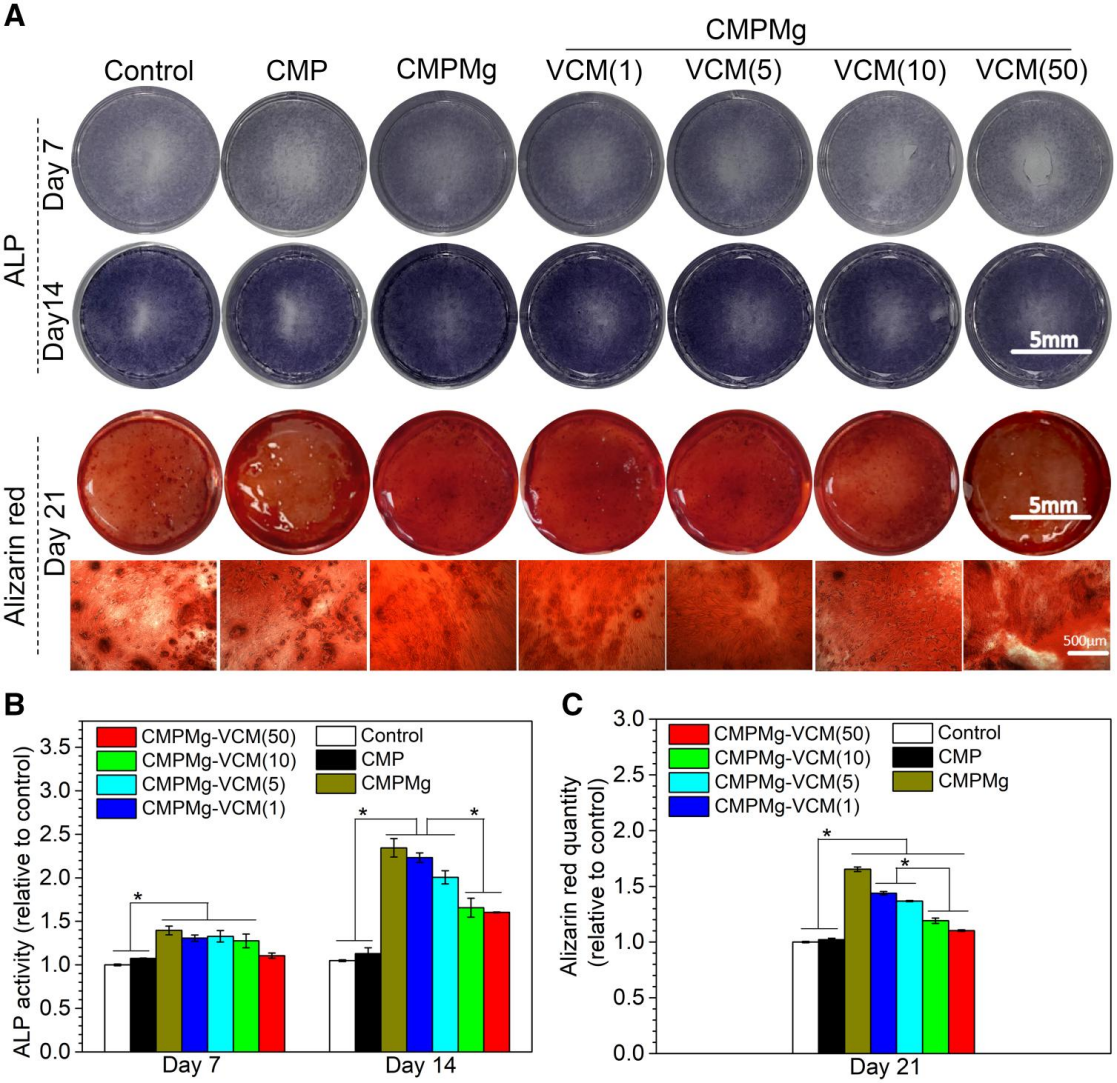
Osteogenic promotive property of hydrogels. (**A**) ALP and alizarin red staining images for the cells when cultured with different samples for 7, 14 and 21 days, (**B**) ALP activity for the cells cultured with different samples for 7 and 14 days and (**C**) alizarin red quantity for the staining (*n* = 4, *P < 0.05).

The MC3T3-E1 cytotoxicity of hydrogels is shown in Figure 7. From the live/dead staining results in Figure 7A, all the hydrogel-cultured MC3T3-E1 cells showed some dead cells after being co-cultured with hydrogel for 3 and 5 days, and the control group showed a lesser number of cells in these two time points. The cell viability results in Figure 7B displayed that only CMPMg and CMPMg-VCM (50) hydrogels showed <75% cell viability, but other samples had >75% cell viability at 3 days of culture, and after 5 days of culture, all the hydrogels showed >75% cell viability. The >75% cell viability indicates no cytotoxicity to this cell, according to the reported study [24]. The *in vitro* osteogenic promotive property of hydrogels was investigated, and the result is displayed in Figure 8. From macroscopical images for ALP staining results, at day 7, the CMPMg, CMPMg-VCM (1) and CMPMg-VCM (5) hydrogels showed a deeper black-brown stained colour when compared with other hydrogel samples and the control group; at day 14, all the MgO nanoparticles contained hydrogels that showed a deeper ALP-stained colour when compared with the CMP hydrogel and the control group. The ALP activity result in Figure 8B showed the same tendency with ALP staining observation, and at day 14, the CMPMg, CMPMg-VCM (1) and CMPMg-VCM (5) showed the highest ALP activity among these samples. The alizarin red staining result for the MC3T3-E1 cells cultured with samples for 21 days showed that all the MgO nanoparticles contained hydrogels and displayed a deeper red colour in comparison with the CMP hydrogel and control group. The statistical analysis result in Figure 8C confirmed the calcium nodule-stained result tendency. All the MgO nanoparticles that incorporated hydrogels showed a higher quantity of stained alizarin red when compared with CMP hydrogel and the control group.

### *In vivo* animal study

#### Bacterial culture assay and micro-CT result

**Figure 9.**
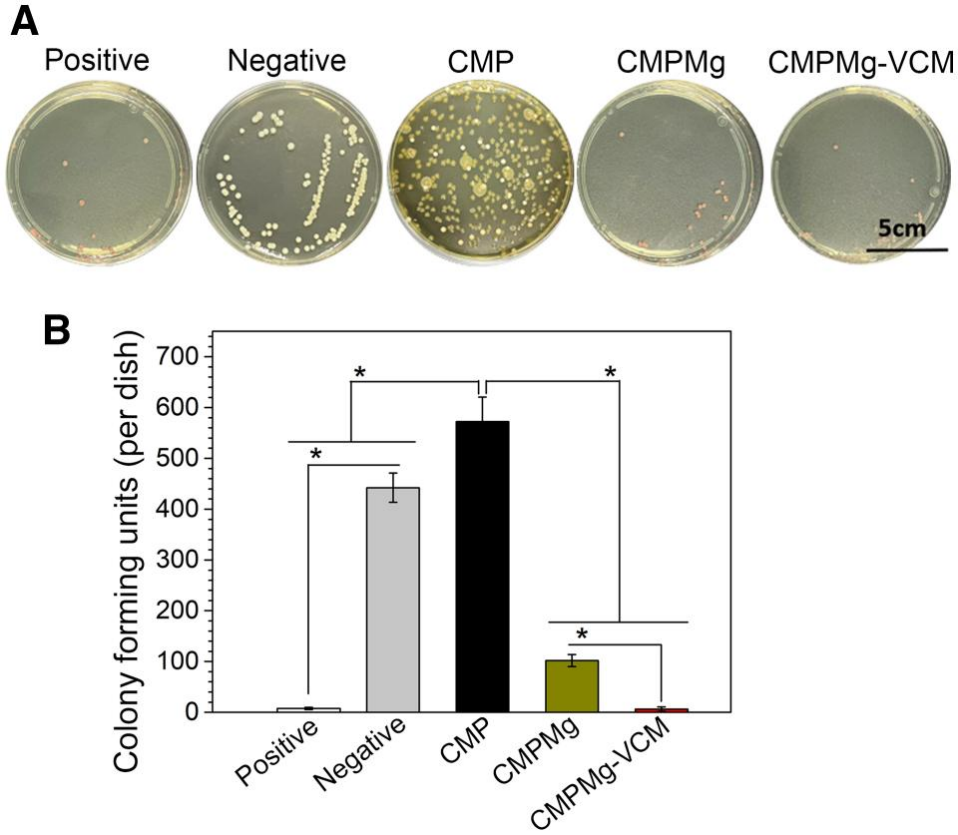
Bacterial culture assay for the tibia after samples were implanted for 6 weeks. (**A**) Macroscopical images for the bacteria colony, (**B**) the statistically analysis result for CFU (*n* = 3, *P < 0.05).

The *in vivo* antibacterial and osteolytic protective properties of hydrogels were investigated by implanting the hydrogels in the intramedullary space of the tibia bone in OM rats. After 6-week implantation, the obtained tibia was immersed in PBS solution, and these PBS solutions were used to perform the bacterial culture experiment. From the bacterial culture result in Figure 9A, the CMPMg-VCM and CMPMg implanted groups showed few scattered bacterial colonies, which was lower than that of CMP and the negative control. Among these samples, the CMPMg-VCM showed the fewest bacterial colonies. The statistical analysis result in Figure 9B confirmed the macroscopical observation in Figure 9A.

**Figure 10.**
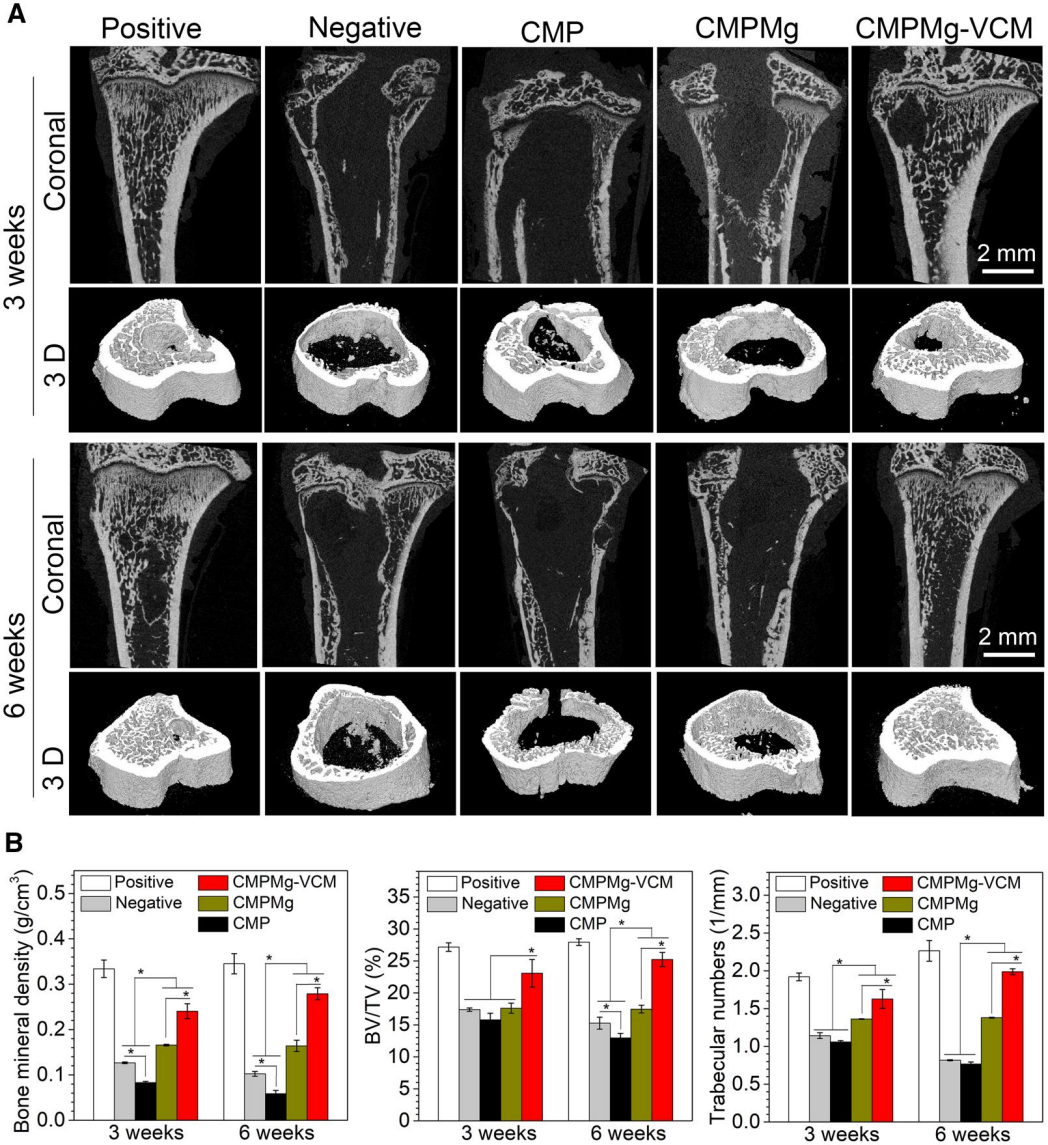
The *in vivo* antibacterial and osteolysis protective property of hydrogels when implanted in the intramedullary space in tibia bone in the OM rat. (**A**) micro-CT data, and (**B**) the statistically analysis results for bone mineral density, bone volume vs. tissue volume ratio and trabecular numbers in the OM area.

Micro-CT on the tibia was performed to investigate the osteolysis protective ability of hydrogels, and the results are shown in Figure 10. After 3-weeks of implantation, in the samples implanted, the CMPMg-VCM hydrogel implanted group showed more bone tissue as compared with CMPMg and CMP hydrogels and the negative control groups. With the implantation time up to 6 weeks, the same tendency was observed in these samples, but no obvious hole could be detected in the CMPMg-VCM group. The CMPMg and CMP hydrogels presented the hole in the region of interest (ROI) area, especially the CMP hydrogel, which almost showed the same hole size with a negative group. The bone parameters in the ROI were analyzed from micro-CT data (in Figure 10B). The bone mineral density (BMD) for CMPMg-VCM was larger than that of the CMPMg, CMP and negative control groups after 3 and 6 weeks of implantation. The ratio of bone volume to tissue volume and the trabecular numbers among these samples showed the same tendency as the BMD result.

#### H&E, Masson and Gram’s staining

**Figure 11.**
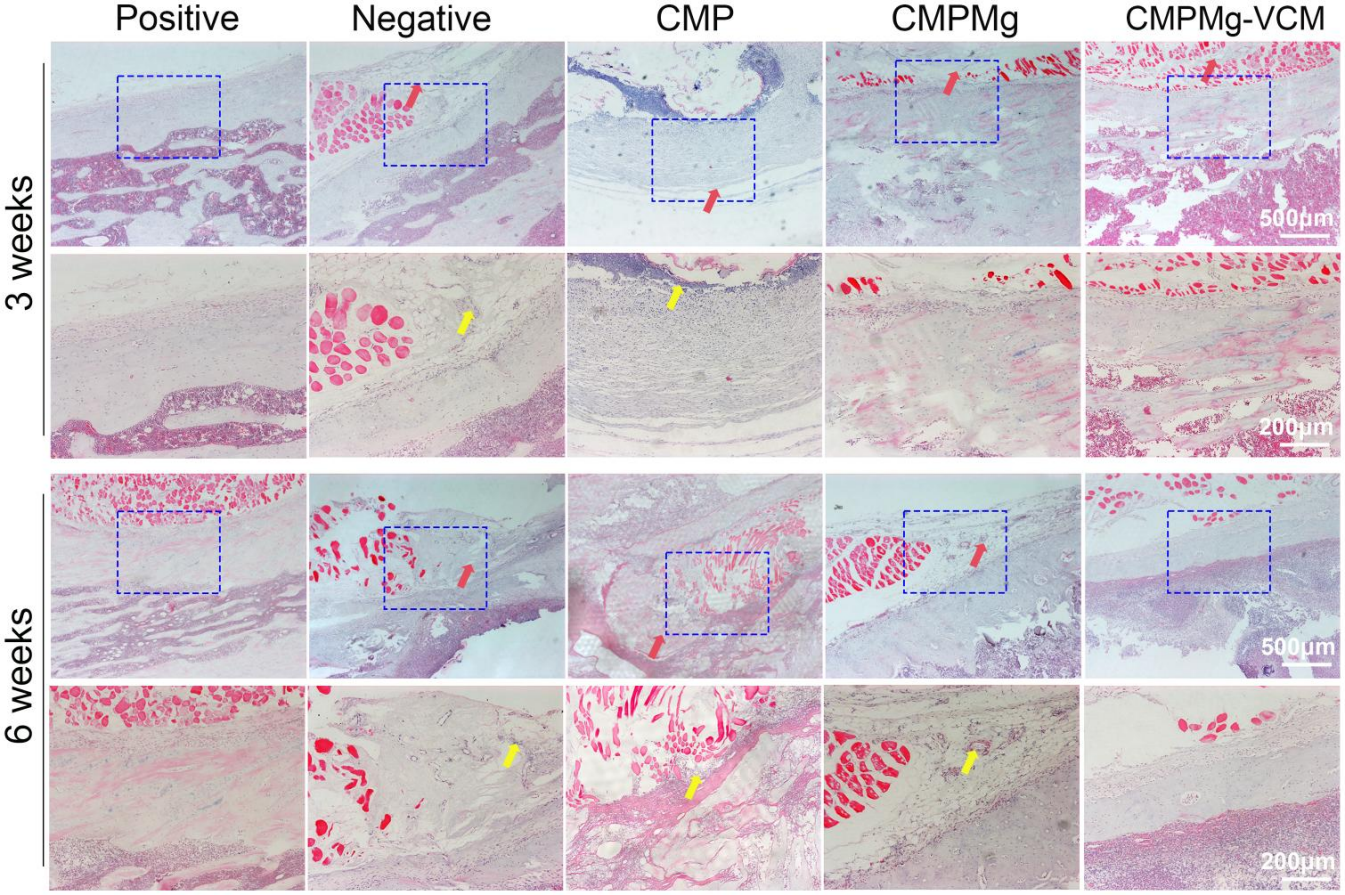
H&E staining results for the infected area in the tibia bone after implanted hydrogels for 3 and 6 weeks. The red arrow stands for bone destruction, including cellular-like and laminar-like structures; the yellow arrow stands for inflammatory cell infiltration.

**Figure 12.**
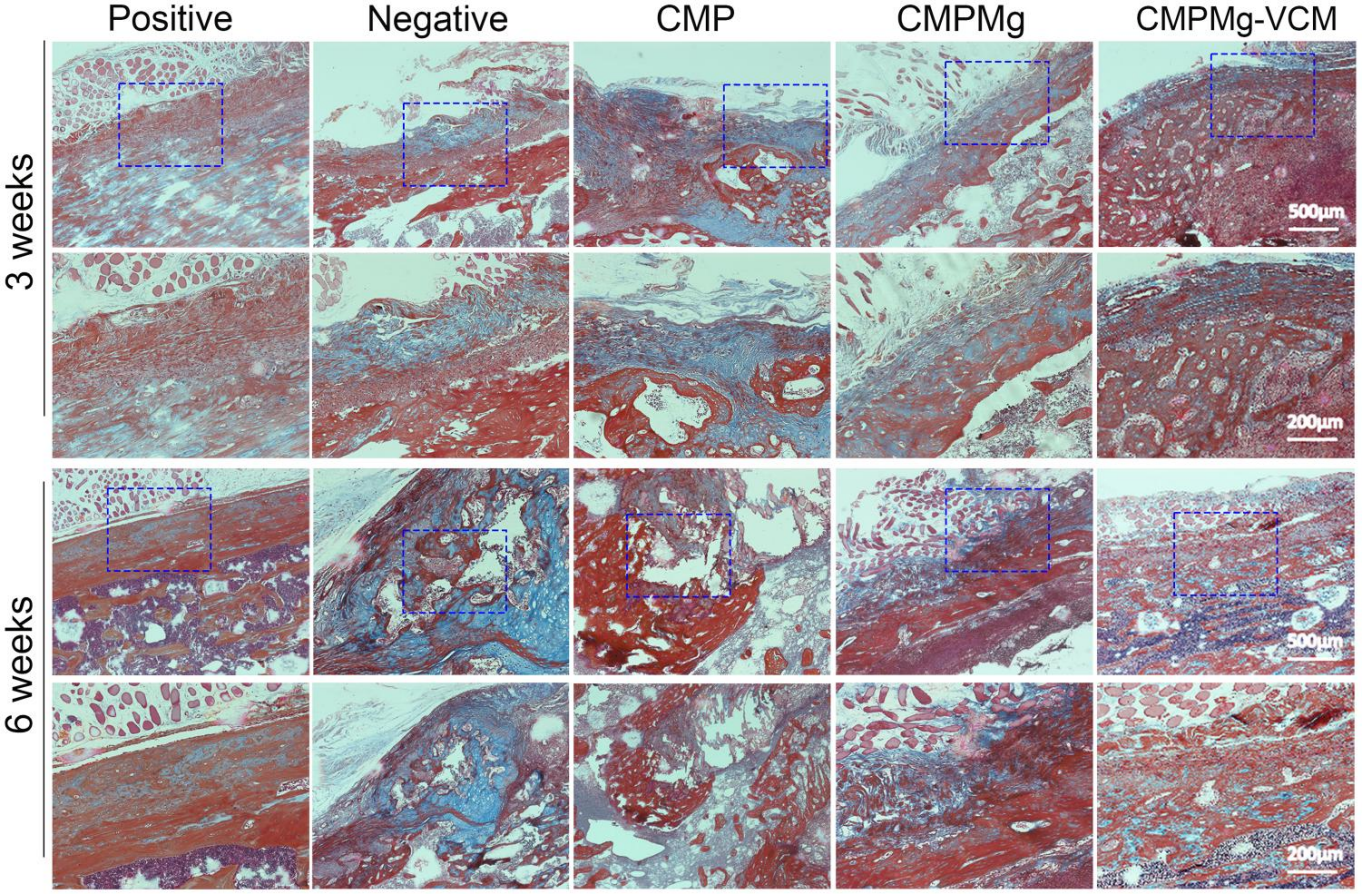
Masson staining results for the infected area in the tibia bone after samples were implanted for 3 and 6 weeks.

**Figure 13.**
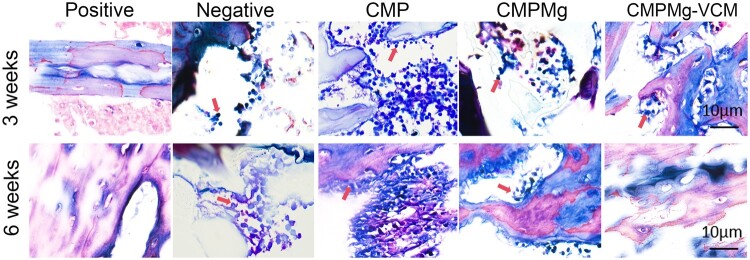
Gram’s staining results for the infected area in the tibia bone after samples were implanted for 3 and 6 weeks. The bluish-violet stained spheroid was the SA (red arrow).

The H&E staining (in Figure 11) results showed that at week 3, there were many bone destruction areas, such as cellular-like structure and laminar-like structure (as marked with a red arrow) in the negative and CMP groups, but after implanting with the CMPMg and CMPMg-VCM groups, especially the CMPMg-VCM group, the bone destruction area decreased. Meanwhile, the inflammatory cell infiltration (blue dots) among these groups showed the same tendency for the bone destruction. At week 6, the bone destruction situation became worse for the negative and CMP groups; especially for the CMP group, most of the bone was destroyed, but the CMPMg-VCM group presented no obvious bone destruction or inflammatory cell infiltration. The Masson staining results in Figure 12 showed the same tendency as the H&E staining results. There was severe bone damage for the negative and CMP groups at these two time points, but the CMPMg-VCM group showed no obvious bone damage after 3 and 6 weeks of implantation. The semi-quantitative histological scoring for the inflammation in the H&E staining result is shown in Supplementary Figure S1. In these four indexes, a high score means low inflammation. The score for CMPMg-VCM was higher than that of the CMP and negative control groups, indicating a low inflammatory reaction. Figure 13 showed the Gram’s staining results, and the bluish-violet-stained spheroid was the SA (marked as a red arrow). All the bacterially injected samples, including negative control, CMP, CMPMg and CMPMg-VCM, presented a bluish-violet-stained spheroid after 3 weeks of implantation, and the CMPMg-VCM implanted group showed the least amount of stained spheroid. After 6 weeks of implantation, the tendency among these samples was the same, but an obvious stained spheroid was presented in the CMPMg-VCM implanted group, which is similar to the positive control, indicating the excellent antibacterial property of SA *in vivo*.

#### H&E staining for organs

After 6 weeks implantation, the organs, heart, live, spleen, lung and kidney were obtained for H&E staining to evaluate the bio-safety of CMPMg-VCM hydrogel in infected tibia and the result was showed in Supplementary Figure S2. The H&E staining result showed that these organs after implanted with CMPMg-VCM hydrogel displayed the same texture as the control group, indicating no bio-safety issue when implanted this CMPMg-VCM hydrogel.

## Discussion

The management of OM is a challenging issue in clinics. As stated in the description, the biomaterials with antibacterial, biodegradable, osteogenic promotive and injectable properties show great promise in OM treatment. In this study, an injectable VCM-loaded magnesium coordinated phosphate CS-based hydrogel was fabricated to treat OM. VCM was dissolved in DI water in a high concentration and then mixed into phosphocreatine-grafted CS water solution, followed by the formation of injectable hydrogels after being mixed with a suitable amount of MgO nanoparticles. The formation mechanism of this hydrogel was the same as in our previous study [12]. To be specific, the Mg(OH)_2_ covered on the surface of MgO nanoparticles was combined with phosphate groups in modified CS via metal-organic coordination, and when the added MgO NPs reached a certain concentration, the spatial network structure based on MgO NPs would be formed. The hydrogel formation condition is simple and mild, and the formation time could be controlled with the addition of MgO NPs. This hydrogel formation is quite different from the reported studies [8].

The included VCM could not affect the formation of hydrogel and could not affect the anti-swelling property of hydrogel in DI water. The low swelling ratio of MgO NPs incorporated into hydrogels might be attributed to that the magnesium hydroxide would combine with protonated amino groups, which would weaken the interaction between water and amino groups in hydrogel, and therefore suppress penetration of water into the hydrogel. In this case, the addition of VCM to this hydrogel could not break the equilibrium state of the MgO NP-incorporated hydrogel system. The low swelling ratio hydrogel has a low volume change ratio after being implanted in the local site, which would not oppress the surrounding tissue, and this is attractive in practical clinical applications. Interestingly, the addition of VCM improved the compressive strength of hydrogel, especially the high amount of VCM (as shown in Figure 1). This might be attributed to that the existing groups, such as amino or hydroxyl groups in VCM, are physically combined with phosphate or other groups in hydrogel, which enhance the hydrogel. As the water-soluble attribute of VCM and the weak physically combined VCM in this hydrogel system, the VCM was released fast in the initial stage during the *in vitro* immersion process (in Figure 4), and the release of VCM was mainly dependent on the diffusion. Due to the high water content and micropore structure of hydrogels, PBS solution or other body fluid in the *in vivo* implantation process penetrated into the hydrogels, which eroded the MgO NPs. MgO could react with water to produce Mg(OH)_2_, which reacts with chloride ions to produce Mg ions and hydroxyl ions, and along with the fluid exchange between hydrogel and surroundings, these ions were released from the hydrogel. The large amount of VCM release could kill the SA and further inhibit the SA colony’s reproduction, which is of importance in OM treatment in clinics. During the OM treatment process, except for the large amount of VCM released in the initial stage to destroy the biofilm, the subsequent bacteriostatic effect was also important to inhibit the reoccurrence of biofilm. Therefore, the antibacterial property of PBS solution immersed with hydrogels for different times was performed (Figure 6); in this assay, at each time point, part of the immersed solution was used for the antibacterial efficacy test, which simulates the *in vivo* implanted microenvironment in which the released VCM would not metabolize so fast at the local site, and this experiment design is similar to the reported study [25]. The excellent antibacterial efficacy of CMPMg-VCM (50) after 12 days of immersion in PBS might be due to that the remaining VCM in the PBS solution would inhibit the release of VCM from the hydrogel, which is different from the VCM release profile in Figure 4, and the released Mg ions and hydroxyl groups produced by the MgO degradation also have bacteriostatic ability.

The CMPMg-VCM hydrogels showed no obvious cytotoxicity to MC3T3-E1 cells and promoted osteogenic differentiation in this cell line, confirming the osteogenic promotive property of this hydrogel system. Only CMPMg and CMPMg-VCM (50) showed <75% cell viability at 3 days of culture, which might be attributed to the high Mg ions concentration and the released VCM. Additionally, this hydrogel showed no bio-safety issue when implanted in infected tibia for 6 weeks (Supplementary Figure S2). No/low cytotoxicity to MC3T3-E1 and no bio-safety issue in the animal study were due to that the released components, including VCM, Mg ions and hydroxyl groups, were in the safe concentration range. The osteogenic promotive property of CMPMg-VCM hydrogel was attributed to the released magnesium ions and alkalescent environment resulting from the MgO degradation, and the released VCM would not affect this process. According to the reported studies [26], the effective concentration of magnesium ions to promote osteogenic differentiation was around 5 mM. Many studies have confirmed the osteogenic promotive property of a suitable concentration of magnesium ions, especially in alkalescent surroundings [27, 28], and the osteogenic promoting mechanism for Mg ions was mainly through Wnt/β-catenin, MAPK and PI3K signaling pathways. Further, the hydrogel was implanted in the SA-infected intramedullary space of the tibia, and the CMPMg-VCM hydrogel killed the implanted bacteria and further inhibited their propagation, which protected the bone from infective osteolysis. The H&E and Masson staining results confirmed that the CMPMg-VCM hydrogel implanted group showed no obvious bone damage, but the CMP hydrogel implanted group showed severe bone destruction (even worse than the negative group). This might be due to that the CMP hydrogel, without antibacterial ability, provided a gentle and comfortable environment beneficial for the growth and propagation of bacteria. After 6 weeks of implantation, the bacterial colony in the CMPMg-VCM group was almost the same as the positive control group (no SA-infected group), and Gram’s staining result also presented the same tendency, confirming no infection in tibia. The appealing antibacterial, osteogenic and injectable properties of this CMPMg-VCM hydrogel in OM treatment could be attributed to the burst release of VCM in the initial stage to destroy the bacterial biofilm, the small amount of VCM and the released magnesium ions and hydroxyl groups to bacteriostatic ability, and the suitable magnesium ions and alkalescent environment for osteogenic promotive properties. This CMPMg-VCM hydrogel showed great promise in OM treatment.

Nevertheless, several points should be mentioned here, especially for further studies. First, there were non-degradable components in this hydrogel system, but total degradation of this hydrogel *in vivo* during the implantation process was not observed. The further study will consider degradation testing, especially *in vivo* animal studies. Second, the hydrogel was implanted in the tibia after the SA was injected (the bacterial biofilm was still not formed), and this animal model is different from OM in clinics. Last but not least, the osteogenic promotive and antibacterial mechanisms of this CMPMg-VCM hydrogel should be systematically investigated in further study.

## Conclusions

In this study, an injectable magnesium-coordinated phosphate CS-based hydrogel loaded with VCM (CMPMg-VCM) was successfully fabricated via a metal-organic coordination strategy. The VCM in this hydrogel system would not affect the formation or anti-swelling properties of the hydrogel. The VCM was released from the CMPMg-VCM hydrogel during the *in vitro* immersion in PBS solution. The CMPMg-VCM hydrogel, especially VCM in high concentration, showed antibacterial properties that killed the SA and inhibited SA growth, and this antibacterial efficacy would last for 12 days during the *in vitro* immersion process. The CMPMg-VCM hydrogels showed no cytotoxicity to MC3T3-E1 cells and would promote osteogenic differentiation for MC3T3-E1 cells in terms of promoting ALP expression and the formation of calcium nodules. Finally, the CMPMg-VCM hydrogels would inhibit the propagation of SA, and kill the SA and protect the infected bone from osteolysis in the OM rat model. Taken together, the appealing antibacterial, non-cytotoxic and osteogenic promotive properties make this hydrogel an alternative biomaterial for OM treatment.

## Supplementary Material

rbae049_Supplementary_Data

## Data Availability

Data will be made available on request.
