# Supplementary material for: An injectable magnesium-coordinated phosphate chitosan-based hydrogel loaded with vancomycin for antibacterial and osteogenesis in the treatment of osteomyelitis
Source: Regen Biomater. 2024 May 25;11:rbae049. doi: 10.1093/rb/rbae049 (PMC11196881; doi:10.1093/rb/rbae049)
Supplement: rbae049_Supplementary_Data [file rbae049_supplementary_data.doc]

**An injectable magnesium coordinated phosphate chitosan-based hydrogel loaded with vancomycin for antibacterial and osteogenesis in the treatment of osteomyelitis**

Peng Zhang^a^, Tiehua Wang^b^, Junyu Qian^a^, Haotian Qin^a^, Peng Liu^a^, Ao Xiong^a^, Anjaneyulu Udduttula^c^, Deli Wang^a***^, Hui Zeng^a**^, Yingqi Chen^a*^

^a^ Department of Bone & Joint Surgery, National & Local Joint Engineering Research Center of Orthopaedic Biomaterials, Peking University Shenzhen Hospital, Shenzhen 518036, P.R. China

^b^ Internal Medicine, Shenzhen New Frontier United Family Hospital, Shenzhen 518031, P.R. China

^c^ Centre for Biomaterials, Cellular and Molecular Theranostics (CBCMT), Vellore Institute of Technology (VIT), Vellore, Tamil Nadu, 632014, India

^*^**Corresponding author 1:** Associate Professor & P.h.D. Yingqi Chen

Department of Bone & Joint Surgery, National & Local Joint Engineering Research Center of Orthopaedic Biomaterials, Peking University Shenzhen Hospital, Shenzhen 518036, PR China

**E-mail**: [yqchen0203@foxmail.com](mailto:yqchen0203@foxmail.com)

^**^**Corresponding author 2:** Prof. & M.D. Hui Zeng

Department of Bone & Joint Surgery, National & Local Joint Engineering Research Center of Orthopaedic Biomaterials, Peking University Shenzhen Hospital, Shenzhen 518036, PR China

**E-mail**: [zenghui@pkuszh.com](mailto:zenghui@pkuszh.com)

^***^**Corresponding author 3:** Prof. & M.D. Deli Wang

Department of Bone & Joint Surgery, National & Local Joint Engineering Research Center of Orthopaedic Biomaterials, Peking University Shenzhen Hospital, Shenzhen 518036, PR China

**E-mail**: [wangdelinavy@163.com](mailto:wangdelinavy@163.com)


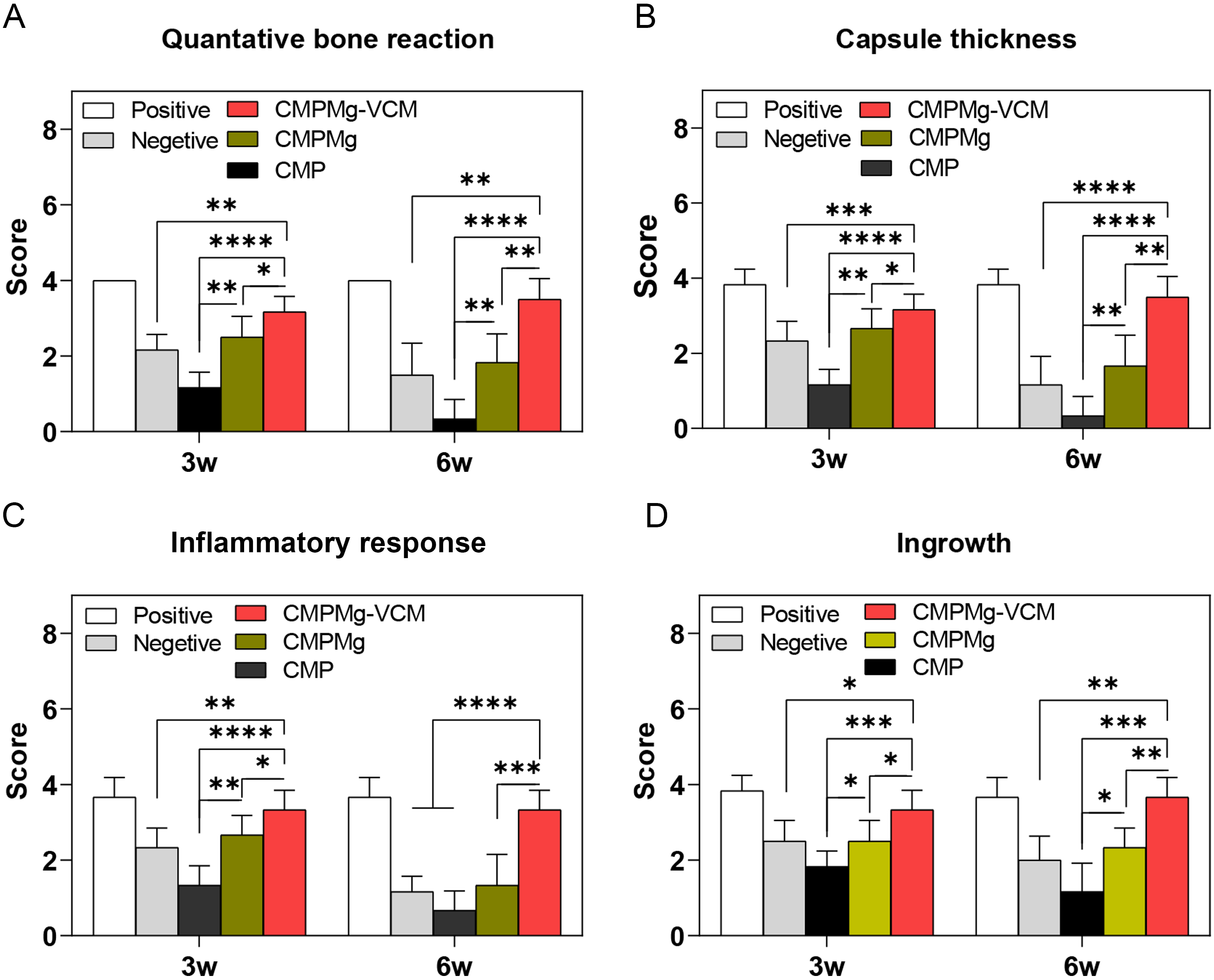


**Figure S1**. The semi-quantitative histology scoring for inflammation calculated from H&E staining. (A) capsule thickness, (B) qualitative bone reaction, (C) inflammation response, and (D) bone ingrowth (n=3, *p < 0.05, **p < 0.005, ***p < 0.0005, ****p < 0.00005).


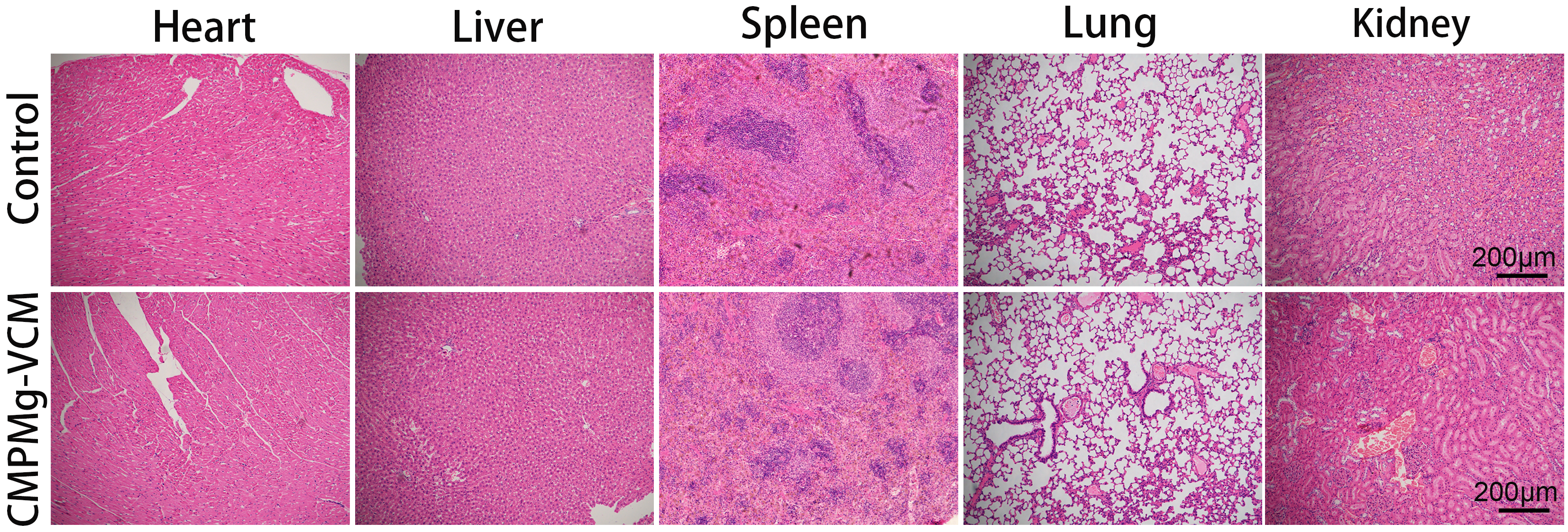


**Figure S2**. H&E staining results for the organs after the hydrogels implanted in the infected area in the tibia bone for 6 weeks.
